# Osteoid osteoma of the acetabular roof: a case report

**DOI:** 10.1186/s13256-016-1016-2

**Published:** 2016-08-24

**Authors:** Youssef Benyass, Bouchaib Chafry, Kaldadak Koufagued, Belkacem Chagar

**Affiliations:** Department of Orthopedic Trauma, Mohamed V Military Hospital, University Mohamed V- Souissi, Rabat, Morocco

**Keywords:** Osteoid osteoma, Percutaneous treatment, Interventional radiology

## Abstract

**Background:**

An acetabular location of osteoid osteoma is rare and represents less than 1 % of cases. The purpose of this clinical case report is to highlight the unusual location of osteoid osteoma and the technical difficulty of its removal.

**Case presentation:**

We report a case of a 17-year-old Moroccan Arab boy who presented with pain in his right hip with lameness. The diagnosis of osteoid osteoma was made by imagery and confirmed by histological examination. The treatment consisted of a complete percutaneous resection scanno-guided of the nidus. The evolution was marked by complete healing with total and definitive disappearance of symptoms after 1 year.

**Conclusions:**

Osteoid osteoma of the acetabular roof is rare. The diagnosis is now easy because of the evolution of imaging. Treatment is exclusively surgical. Complete resection guarantees the absence of recurrence. The difficulty of the surgical procedure is due to the deep localization of the osteoid osteoma and because it is endosseous, it is also dangerous due to anatomical relationships and the small size of the osteoid osteoma.

## Background

Osteoid osteoma is a common benign bone tumor. It represents 10 to 12 % of all benign bone tumors [1]. In most cases it occurs in the first three decades of life with a male predominance. It occurs primarily in the lower limbs. Acetabular involvement is very rare (≤1 %) [2]. Imaging is the key to diagnosis. Treatment of osteoid osteoma is exclusively surgical. Complete resection guarantees an absence of recurrence. If the localization of the osteoid osteoma is deep, then the surgical procedure is difficult. The purpose of this clinical case report is to highlight this unusual site of osteoid osteoma and the technical difficulty of its removal.

## Case presentation

**Fig. 1:**
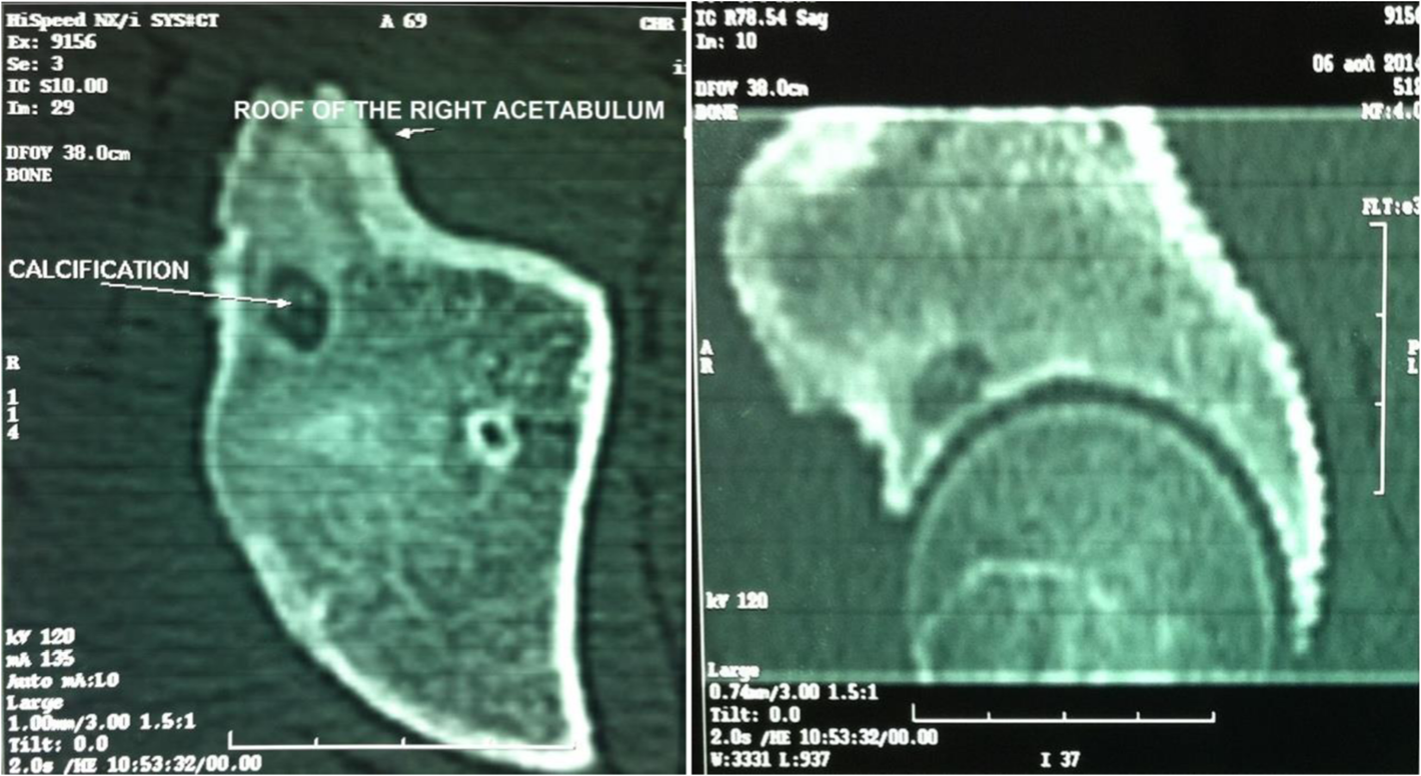
Computed tomography scan showing an osteoid osteoma at the acetabular roof

A 17-year-old Moroccan Arab boy without significant medical history presented last year with pain in his right hip that was predominantly nocturnal and sensitive to aspirin. A clinical examination showed lameness while walking without limitation of hip movements. The results of laboratory tests were normal. Plain radiographs of his right hip did not show any visible lesion. A computed tomography (CT) scan showed a 10 mm osteoid osteoma located in the roof of the right acetabulum (Fig. 1).

**Fig. 2:**
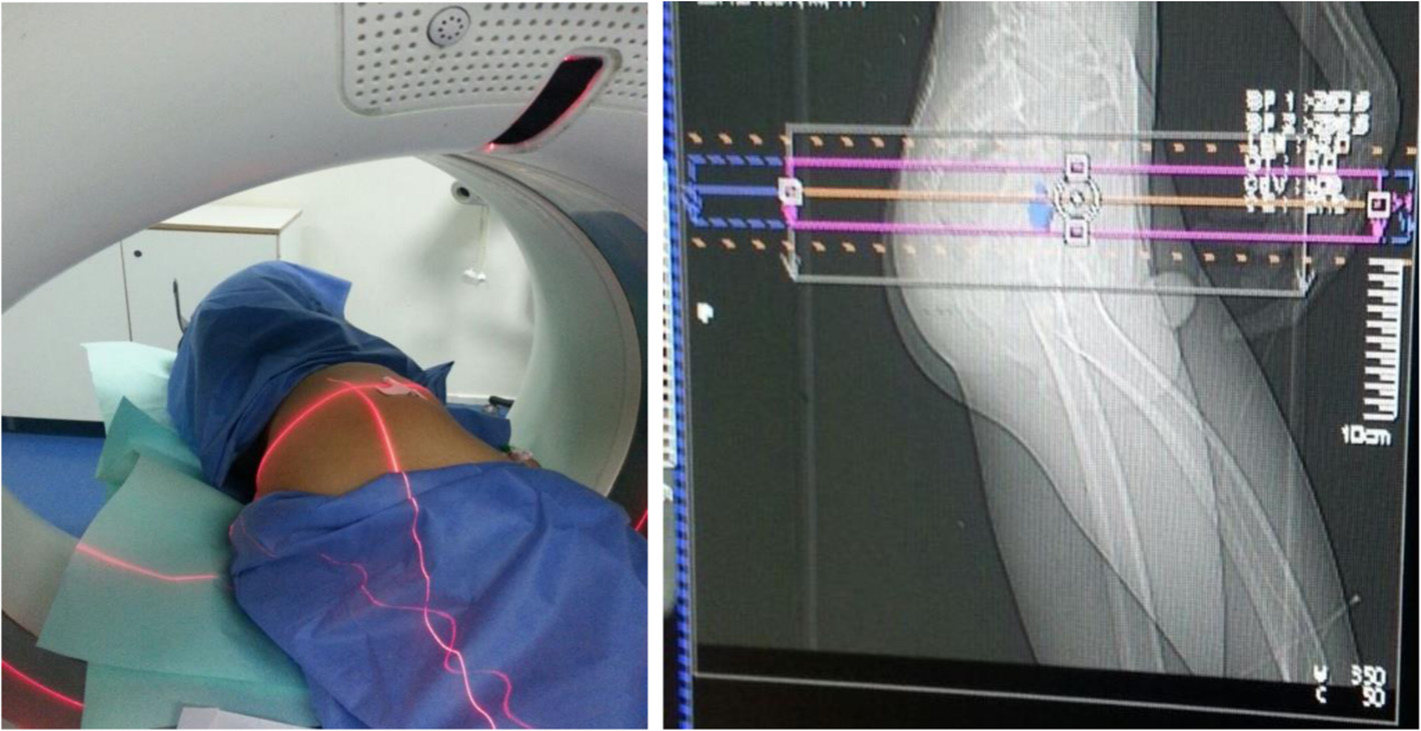
Locating osteoid osteoma by contiguous computed tomography slices

**Fig. 3:**
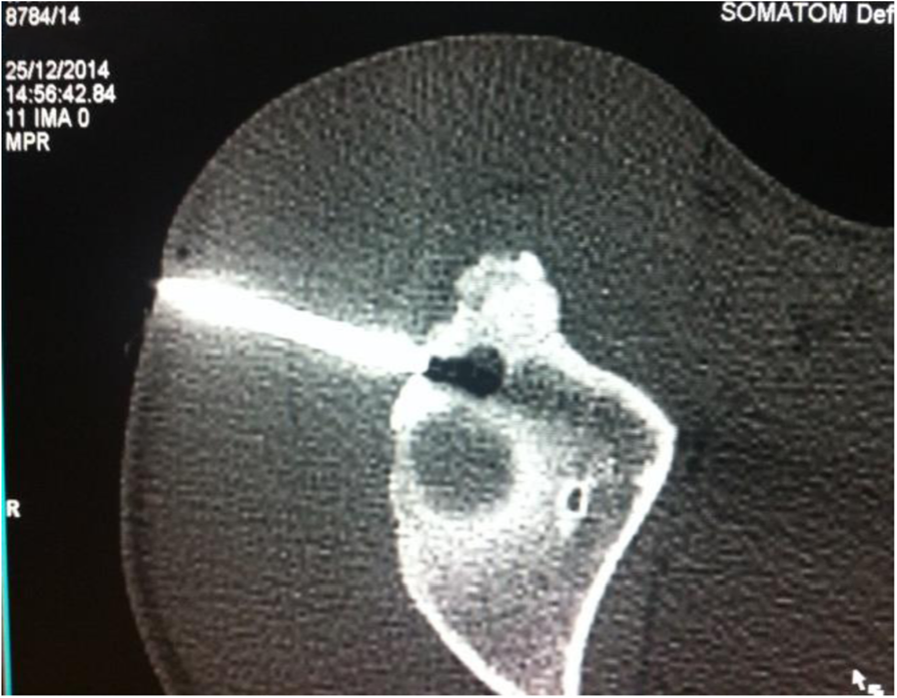
Computed tomography control immediately after resection confirmed the complete resection of osteoid osteoma

He was operated in the radiology department with surgical aseptic conditions, under spinal anesthesia and in the lateral decubitus position. The lesion was identified by contiguous 2 mm thickness CT scan (Fig. 2). Then the nidus was extracted with percutaneous curettage using a trephine. A CT scan immediately after resection confirmed complete resection (Fig. 3). Histological examination confirmed the diagnosis.

After a period of 1 year, evolution was marked by a complete recovery with total and definitive disappearance of symptoms.

## Discussion

Osteoid osteoma is a benign bone tumor of small size, described for the first time by Jaffe in 1935. It represents 10 to 12 % of benign bone tumors; it usually affects individuals during the first three decades of life, with a predominance in males [3]. It is characterized by the presence of a central hypervascular nidus which may calcify. It is formed by an amalgamation of mineralized osteoid spans whose size does not exceed 15 mm. An osteosclerosis reaction of varying size surrounds the nidus.

Osteoid osteoma preferentially localizes at the shaft of long bones near the metaphyseal junctions, with a predilection for the lower limbs [4]. It is located very rarely on flat bones. Clinical manifestations are usually typical, such as night pain with insomnia, relieved by aspirin taking. In our case, the symptoms were typical and associated with lameness. This pain is related to high levels of prostaglandins in the nidus, which induce an inflammatory response [5]. A clinical examination is usually normal.

Conventional radiographs can objectify intracortical lacuna, containing sometimes a punctuated matrix surrounded by a sclerotic reaction more or less important. In joint juxta articular forms, the peripheral condensation is low and there is often a synovial thickening revealing intra-articular effusions, which can mislead the diagnosis [6]. In the case of our patient, plain radiographs did not objectify the injury. Bone scintigraphy is indicated in front of an evocative board confirming a suggestive image on plain radiographs or detecting lesions in normal radiographs. It shows an early spot, very hyperfixant that is characteristic of the nidus surrounded by a less intense hyperfixing area. It is very sensitive but not specific [7].

CT has significantly increased the speed of diagnosis as the early clinical signs appear. It also allows you to locate precisely the lesion and the exact size of the nidus. It is the gold standard provided thin sections of 1 to 2 mm thickness are used [8]. CT in our patient confirmed the diagnosis of osteoid osteoma. It showed the presence of a spongy 10 mm lacuna at the roof of his right acetabulum, surrounded by an osteosclerosis reaction, with the presence of central punctuated calcifications.

The use of magnetic resonance imaging (MRI) is justified especially in cases of doubt in the diagnosis as in unusual localizations, such as juxta-articular or intramedullar. It shows not only the nidus but also the inflammatory reaction modifications in bone marrow and surrounding soft tissue. The use of dynamic sequences after gadolinium injection improves the sensitivity of MRI (92 %) so much that it becomes equal to that of CT [9].

Treatment of osteoid osteoma is surgical. Bloc resection has been the standard therapy. However, difficulty in locating the nidus, despite using preoperative or perioperative imaging, results in a wide bone resection compared to the small size of the lesion. Open surgical treatment is effective when the nidus is resected completely, but remains a relatively invasive technique which weakens the bones, especially long bones, which can justify resorting to a bone graft or internal fixation osteosynthesis and postoperative immobilization [10]. The percutaneous treatment of osteoid osteoma has gradually replaced surgery due to advances in CT and development of percutaneous surgery tools. They are applicable in the vast majority of locations of osteoid osteoma. They allow intraoperative control of needle placement with high accuracy, lower complication rates, faster recovery, and probably a reduction in recurrences [11].

Percutaneous resection under CT guidance is an effective technique and noninvasive. It is practiced in the scanner room under strict aseptic conditions and under local or general anesthesia. It is useful especially in deep locations such as the acetabulum because it avoids dislocating the hip for a direct approach. This procedure uses a coaxial system comprising a guidewire, a trocar mandrel and a toothed outer sheath to protect the soft tissue, a cannulated drill bit to penetrate the bone to the edge of the nidus in the case of a deep lesion, and a trephine of 3 to 8 mm in diameter for the excision of the nidus. Some use a motor to move the wick and the trephine.

A CT control checks the total resection of the nidus. In a case of incomplete excision, a new borehole can be done. Histological evidence of the presence of a nidus within the resected bone core is reported in 50 to 100 % of cases depending on the series [12].

Other less invasive alternatives have been developed, mostly under CT guidance, comprising alcoholization, thermocoagulation by radiofrequency, laser photocoagulation, and cryotherapy. Currently, surgery is reserved for percutaneously inaccessible locations (posterior wall of the spinal lesion or root canal) or locations adjacent to neurovascular structures (<1 cm) [11].

From the first postoperative hours, the virtual disappearance of pain or complete attenuation supports the use of a total resection. Pain disappears after 24 hours in most cases, but can sometimes persist for a month [13]. The diagnostic approach to a possible recurrence or persistence of an osteoid osteoma is particularly delicate because the surgical act transforms the tumor site. Except in the absence of osteosynthesis material, CT is still the key examination in seeking a “forgotten” nidus in the affected zone [14].

## Conclusions

Osteoid osteoma is a small benign bone tumor. It is rarely located on the acetabulum. CT is the gold standard for diagnosis. The use of MRI is justified if there is doubt in the diagnosis especially in unusual localizations, such as juxta articular or intramedullar forms. Treatment of osteoid osteoma is exclusively surgical. Percutaneous resection under CT guidance is an effective technique, especially in the forms with deep anatomical location, where it is extremely efficient. It allows perioperative control with high precision, lower complication rates, faster recovery and probably a reduction in recurrences. Several procedures and techniques for treating osteoid osteoma percutaneously are currently available to the clinician. Surgical treatment is reserved for percutaneously inaccessible locations or locations near neurovascular structures.

## Abbreviations

CT, computed tomography; MRI, magnetic resonance imaging
